# Geopsychiatry from below: Exploratory review and preliminary analysis

**DOI:** 10.1177/00207640251317017

**Published:** 2025-02-24

**Authors:** Cheryl McGeachan, Chris Philo

**Affiliations:** School of Geographical & Earth Sciences, University of Glasgow, UK

**Keywords:** geopsychiatry, ‘the geo’, place and space, voices of experience

## Abstract

**Background:**

This contribution advances claims about ‘geopsychiatry from below’, attending to how ‘voices’ with lived experience of mental ill-health speak about ‘the geo’ or, more specifically, ‘place and space’.

**Aims:**

To explore relevant interdisciplinary literature for academic research, scholarship and commentary containing voices of experience speaking about the geo.

**Methods:**

An ‘indicative’ and ‘facilitative’ review of relevant transdisciplinary literature in arts and humanities and social science, alongside an exploratory workshop where materials are analysed and relationships detected and, provisionally, mapped.

**Results:**

The literature review discloses no coherent body of studies into the geo from below, but rather a fragmented amalgam of materials—field observations, primary quotes and occasional elaborations—that are rarely the direct focus of inquiry (except in some contributions by academic geographers). Combining the literature review and the workshop analysis, an outline series of prompts are developed for relating ‘Kinds of Places’, their associated ‘Affective Qualities’ and actual spaces and places on the ground.

**Conclusions:**

This study signals what a geopsychiatry from below might entail, providing important foundations for future transdisciplinary work on ‘the geo’ and mental (ill-)health.

## Geopsychiatry from below?

There has recently been a growing interest in what has been called ‘geopsychiatry’ (Alibudbud, 2024a, 2024b; Bhugra & Ventriglio, 2023a, 2023b, 2023c; Castaldelli-Maia & Bhugra, 2022; Koravangattu et al., 2022; Persaud & Bhugra, 2022a, 2022b, 2022c; Persaud et al., 2018; Persaud & Ventriglio, 2023; Smith et al., 2023; Sri et al., 2023; Valsraj et al., 2022), with the field emerging as an insightful point of interconnection between psychiatry, as academic discipline and clinical practice, and geography, as academic discipline and a ‘sensibility’ hinging on acknowledging the importance of ‘space and place’ (and a host of related constructs such as environment, landscape, location and distribution).

To date, inquiries framing themselves as geopsychiatry have addressed the global scale of regional disparities in ‘determinants’ threatening to mental well-being. This focus directs attention to the global theatres of: geopolitical tension, violence against ethnic and other minorities, and the forced migration and struggles of refugees and aslyum-seekers; geoeconomic inequalities between world regions bound up with the uneven workings of capitalism, neo-colonialism and ‘development’; and, to a lesser extent, geocultural dimensions of how powerful societal cohorts discriminate against ‘minority’ groupings on the grounds of race (ethnicity), gender, sexuality and other (innate or attributed) characteristics. Additionally, concern turns to the enormous environmental challenges currently biting for many peoples and places, prompted by climate change, extreme weather events, flooding, desertification, land erosion and more, all impacting the ‘security architecture’ of individuals and communities worldwide. Questions of geographical variability in policy-and-practice responses to situations producing mental ill-health are also woven into these early efforts at founding geopsychiatry.

There is no doubting the value of such inquiries, although we might wish for more credit to be given to existing work in academic geography—our home discipline—that, occasionally explicitly conducted under the umbrella of ‘mental health geography’, tackles these sorts of matters. Such was the message of a piece that we published in this journal—with two other colleagues—on the theme of ‘geopsychiatry and geography’ (Philo et al., 2024). A key feature was to underline that mental health geography has taken seriously critiques of ‘the biomedically-inflected model of illness, pathology and clinical intervention typical of medical geography’ (Philo et al, 2024, p. 82). As such, we have reservations about straightforwardly combining geography with more biomedical versions of psychiatry, but also anticipate that our favoured orientation will dovetail with the ‘social psychiatry’ integral to the ethos of this journal and, moreover, with efforts already made in certain quarters of psychiatry to allow the views of ‘patient’ representatives and advocacy groups to influence both academic research and clinical practice.

In our discussion, moreover, we emphasised how mental health geographers often strive to appreciate ‘the ‘internal’ or ‘agentic’ worlds of people who acquire mental health problems,’ showing ‘commitment . . . to engaging with the lived experience of mental (ill-)health’ (p. 83). Another way of expressing that remark is to claim the value of what here we will term ‘geopsychiatry from below’,^1^ centralising voices of experience—emanating from people who live with or are recovering from mental ill-health—and what they may represent, directly or in passing, about their lived experiences of ‘the geo’. We coin the latter term, meanwhile, as a shorthand for ‘space and place’ and related constructs, each of which can be lent precise (if contested) definition by academic geographers but are, unsurprisingly, for the most part used quite imprecisely and interchangeably in everyday life settings.

Centralising voices from below is one way whereby we distance ourselves from a mainstream of psychiatric thought-and-practice that does still tend to regard ‘patient’ views, opinions and experiences as merely expressing—maybe diagnostic of—mental disorder, not necessarily to be engaged for fresh, meaningful insights.^2^ In so doing, we work in solidarity with ‘Mad Studies’ that continue to push for a prioritisation of voices from below (Beresford & Russo, 2022, 2023) and we consult the *International Mad Studies Journal* in what follows. Geopsychiatry, named as such, is nonetheless only a very recent invention, making it premature to conclude that no room can be given such voices here or to suppose that their relative absence as yet necessarily stems from theoretical, methodological or ethical stances already engrained in the field’s ‘DNA’. Indeed, the field is still very much an open book with the potential to be written in many different ways.

The purpose of the present paper is hence to break the ground for an envisaged ‘geopsychiatry from below’, and to do so through the vehicle of a transdisciplinary literature review coupled to a workshopped analysis of materials obtained from that review. This work has been undertaken by the two authors together with a ‘professional storyteller’, Ruth Kirkpatrick,^3^ and a ‘lived experience consultant’, Morag Macgilchrist.^4^ The combined outcome from the review and the workshops has resulted in what we cast as a preliminary framework—or, more humbly, a series of prompts—for detecting inter-relationships within these materials between voiced ‘affective’ attributes of the geo, on the one hand, and real worldly environments and landscapes, on the other. In what follows, we first clarify the methods that we have used in our inquiry, before exploring our results—reflecting on what the literature review has disclosed—and then recounting what emerged from our workshopped analysis in the form of a framework or prompts. In conclusion, we venture final thoughts about the kind of departure that geopsychiatry, and specifically geopsychiatry from below, entails.

## Methods

### Search strategy and procedures

Our inquiry pivots around a literature review. Our background in mental health geography means that we are already familiar with writings in the literature of academic geography wherein voicings of the geo from below are prominent (McGeachan & Philo, 2017, 2023), and we have included two widely-read geography journals in our review and analysis. Our prime interest here, however, is excavating a wider transdisciplinary terrain including both traditional disciplines (e.g. anthropology, history) and interdisciplinary fields (e.g. public health, Mad Studies). Given our interest in paying acute attention to the geo from below, we are attuned to ideas about alternative pathways to knowledge and looking in the margins (Burstow et al., 2014). A prior supposition is that materials germane to our inquiry will be found in certain corners of the transdisciplinary terrain, leading us to sample nine academic journals as the basis for our review (see Table 1). We make no great claims about the status, representativeness or comprehensiveness of this selection, except to note that we have prioritised journals leaning away from the natural sciences—the medical sciences included—and instead toward the social sciences and arts and humanities. Such a leaning is consistent with the distance that we hold between our version of geography and a biomedically inclined psychiatry (excepting that elements of the latter feature in one of our selected journals, the *International Journal of Social Psychiatry*).

**Table 1.** Keywords journal search (numerical search conducted 09/08/2024).

Journal	Mental health and place	Madness and space	Mental health geography	Notes on findings from journal search
*Annals of the American Association of Geographers*	782 ^a^ [0] ^b^	143 [0]	784 [2]	Relatively few papers specifically on ‘mental health’ topics, but does locate all those—including a few carrying voices of experience speaking of the geo from below—as previously sourced by us from this journal (McGeachan and Philo, 2023).
*Health and Place*	1326 [12]	29 [0]	1202 [18]	Many of these papers specifically on ‘mental health’ topics, with 50+ carrying voices of experience speaking of the geo from below.
*History of Psychiatry*	1,262 [0]	395 [0]	176 [0]	‘Madness’ features more than elsewhere because of its ubiquity in the historical record; most papers draw on archival sources where voices of experience (eg. of ‘patients’) are, unsurprisingly, absent or heavily mediated.
*International Journal of Social Psychiatry*	3,548 [1]	88 [0]	594 [1]	Most papers reporting quantitative studies (cohort, survey, longitudinal), conducting policy analyses or providing broadbrush commentaries, so voicings from below, while certainly present, are still relatively limited.
*International Mad Studies Journal* ^c^	n/a	n/a	n/a	All papers concerned with or actively derive from voices of experience.
*Journal of Mental Health*	2364 [0]	43 [0]	341 [2]	A wide diversity of papers relating to varying aspects of mental health. Voices of experience are present in a considerable number of papers but largely mediated through the lens of bio-medical approaches to mental health.
*Medical Anthropology*	490 [0]	83 [0]	141 [0]	Most papers reporting ethnographic inquiries where voices of experience are not directly quoted but close observations are given of how people with mental health problems (‘madness’) appear to be understanding, making meanings and acting in the world.
*Social Science and Medicine*	6384 [12]	131 [0]	87 [11]	Locating a significant number of papers not on ‘mental health’ topics, but still detecting a substantial number (c.50) including voices of experience.
Social and Cultural Geography	430 [0]	100 [0]	432 [1]	Relatively few papers specifically on ‘mental health’ topics, but does locate a small number (c.20) where voices of experience speak of the geo from below.

a Numbers without brackets indicates result from a basic keyword search that finds all papers where the words in the search term (i.e. mental health geography) appear but not necessarily together. Experimenting with Boolean operators does not meaningfully change such results.

b Numbers in brackets indicates result from a keyword search with the search term in double quote marks (i.e. “mental health and place”) that only finds papers where the words in the search term appear together.

c This journal does not offer a keyword search option. Only two issues have been published to-date.

What we conduct might be termed an ‘unsystematic review’, departing from the recent logic of systematic review and its drive to continually narrow down what is to be read. This ‘unsystematic’ approach connects our attempts to excavate ‘from below’ to wider critiques of systematic review processes in Mad Studies and beyond (Rose, 2021). Systematic review uses the technology of repeated online keyword searches and ‘eligibility’ protocols progressively to whittle away the excessiveness of chosen literature databases in extracting only those published papers dealing *exactly* with what interests a researcher (e.g. Mengist et al., 2020). Such an approach is unsuitable for us, its reductionism likely stripping out much of relevance for our purposes and militating against what we position, cryptically, as insatiable curiosity about ‘the next paper’ that might be stumbled across in those more unstructured and serendipitous efforts sometimes called ‘narrative review’ (Gregory & Denniss, 2018).

That said, we acknowledge that some basis is needed to guide our inquiry, and so for each selected journal we have commenced with simple compound keyword searches. Discussion with our lived experience consultant on the project conveyed the importance of attending to the simple and clear coordinates of ‘mental health and place’, ‘madness and space’ and ‘mental health geography’ for our search. Often these searches in any one journal return the same papers, but there are intriguing differences, notably in how including ‘madness’ locates papers with a conceptual, qualitative and critical character often missed by the other search terms. It is possible that papers of potential relevance to us remain unidentified by these searches, but our method does not necessitate exhaustivity and instead simply endeavours to be ‘indicative’ of the sorts of materials that might be located and then ‘facilitative’ of our subsequent analysis.

Table 1 shows the numbers of results returned by these searches for each of our selected journals, showing the dramatically different results from both expansive and more restrictive versions of the searches (see notes under table).^5^ Such enumerations are actually of little significance for our purposes, since the real work begins when we encounter the located papers by reading their titles, abstracts and, if warranted from title and abstract, their text. The key consideration at this stage is whether a paper contains or draws upon—as a core aspect or just in passing—voices of experience that tell us something about living in, with or through the geo. Accessing such voices requires a move away from ‘inclusion criteria’ and quantification of retained papers, and instead requires investigation into the inner parts (depth) of the works themselves, thereby requiring alternative forms of attention. Additionally, where large numbers of papers have been returned by a journal under a search, we have only read titles, abstracts and text for the first circa 50-70 or so of these papers listed in the search results. Again, such non-systematicity is not a problem for us, since our goals are only to arrive at papers carrying materials that are indicative (of what *can* be found in the literature) and facilitative (of *further* work by the authors on the materials located).

### Analysis and workshop design

A further methodological step entailed seeking to convert our findings from the literature review into an analytical—although ‘interpretative’ might be a more accurate term—framework within which certain consequential inter-relationships between place and mental (ill-)health can be specified. Through the medium of a workshop—prepared for by both authors and ran by McGeachan with the lived experience consultant and professional storyteller—extracts selected from the literature review, chiefly direct quotations from voices of experience where the geo was obviously foregrounded,^6^ were carefully considered for what they potentially disclose about how place and mental (ill-)health play upon one another. Connecting to participatory action research (PAR) methods that valorise giving space and voice to lived experience (Billies et al., 2010; Sexton & Sen, 2018), the workshop treated each extract as a vital fragment of such experience, each being engaged both in its particularity and for what it might suggest with generalisable implications.

**Figure 1.**
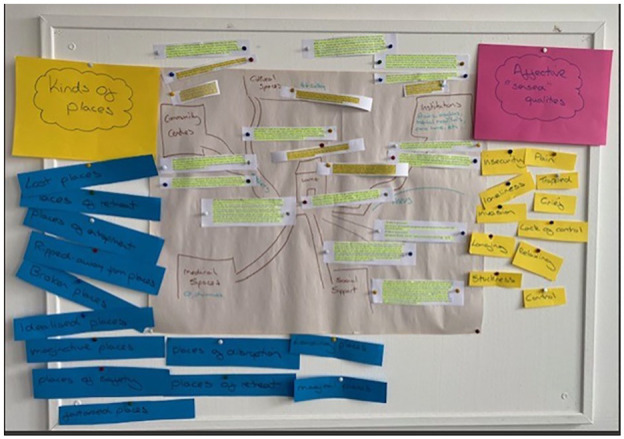
Workshop pinboard (created 28/06/2024).

There were two dimensions of what followed. First, and most analytically, we arrived at lists of ‘Kinds of Places’ and ‘Affective Qualities’ drawing on the literature extracts—we provide thumbnail definitions of what we mean by these terms in Table 2—and we contemplated throughout the workshop how the former, the ‘Places’, are encountered, sensed, interpreted and responded to through the lenses of the latter, the ‘Qualities’.^7^ The second was to ‘map’ the constructs identified under both ‘Kinds of Places’ and ‘Affective Qualities’ in actual spaces (types of location) and concrete places (nameable localities). We used the simple device of a hand-drawn base map which in our workshop identified ‘home’, ‘cultural spaces’ (e.g. art gallery), ‘community centres’, ‘social support (spaces)’, ‘medical spaces’, ‘institutions’ (e.g. hospitals and care homes) and the routes (streets, transport links) between them.^8^ In the workshop we experimented with pinning on to the base map some of our review quotes from voices of experience, ones where clearly identifiable and delimited spaces and places were obviously mentioned or insinuated. The two methodological steps explained here were graphically captured in our traditional workshop pinboard (see Figure 1).

**Table 2.** Elements in the analysis.

Element	What is entails
‘Kinds of places’	Hinging on short descriptors of what spaces and places encompass or represent for people with lived experience of mental ill-health, suggestive of feelings—emotional responses—elicited by the places in question.
‘Affective (‘Sensed’) qualities’	Emphasising overarching affective states for people with lived experience of mental ill-health, rooted in their mental health conditions and biographies, and weighing heavily on what ‘sense’ they make of their world (both their overall existence and the local life-worlds in which they dwell, think and act).
Grounded spaces and places	Meaning the actual spaces (types of location: e.g., park, clinic) and places (named localities: e.g., East End, Glasgow) encountered and occupied by people with lived experience of mental ill-health.

## Results

### Exploring the journal papers

The majority of papers returned by our initial searches with some alertness to the geo do *not* draw upon voices of experience, but rather offer variations on the following: quantitative-statistical analyses of cohort- or population-level mental health data wherein individuals are aggregated into collectives and experiences buried within correlations or purported causal relationships; social and mental health policy analyses majoring on questions about systems, institutions, governance, social change, health outcomes and the like, with only occasional reference to the views of ‘users’; and more abstracted or polemical think-pieces tackling issues in the history or current politics of mental (ill-)health, psychiatry and their contestation, sometimes with the well-being but rarely the actual voices of people with mental health problems at their heart. There is nonetheless a substantial minority of papers—certainly exceeding 100 across our whole sample—where the geo is voiced by people with lived experience, most obviously in direct quotations from these people themselves derived from interviews and focus groups, conducted face-to-face or virtually, or extracted from questionnaires with ‘open questions’ soliciting ‘comments’. In a few cases, notably in observations from ethnographic work, the staple of *Medical Anthropology*, ‘speech’ from people with lived experience is recounted alongside interpretations of motives, meanings and actions. Crucially, a small number of these papers, particularly but not exclusively in the *International Mad Studies Journal*, are the writings of ‘experts’ by experience, hence carrying their voices essentially unfiltered.^9^

For the most part remarks about the geo in our located papers feature indirectly, fleetingly present within broader reflections on other matters such as experiences of treatment regimes, mental health professionals, welfare systems, housing conditions and more. In a few papers, circa 30, these remarks do get foregrounded, in that the direct focus of attention is how people with mental health problems experience—perceive, conceive and represent—diverse spaces and places central to their biographies and ongoing lives. Unsurprisingly, given the overall *raison d’être* of *Health and Place*, it is the journal carrying the richest seam of papers along these lines. While this journal has become a thoroughly interdisciplinary site for a wide-ranging portfolio of public health research, it initially grew from the efforts of academic medical or health geographers (Moon, 1995) and it is the venue in our review where ‘mental health geography’, named as such, appears most frequently.

What is also apparent across our set of located papers is the extent to which voicings of the geo from below—sometimes explicitly citing what is perhaps health geography’s most influential conceptual offering to the transdisciplinary field of ‘social’ health studies, ‘therapeutic landscapes’ (Gesler, 1992, 1993; Kearns & Milligan, 2020)—encompass ‘physical’, ‘social’ and ‘symbolic’ properties of places and spaces. Often overlapping, rarely neatly separated out, voices hence tell of: the physical (scenic, tangible, graspable) aspects of both natural and built environments; the social aspects entailed by human groups, dynamics, relations, inequalities and struggles associated with given locations; and the symbolic, perhaps we might also say cultural, dimensions of what certain spaces and places signify, evoke and impress—what they feel and mean—for the people concerned. Paralleling how the therapeutic landscapes concept has been reworked (e.g. Conradson, 2005), moreover, few if any of our papers imply some innate, fundamental ‘structure’ of space or ‘essence’ of place determining states of mental well- or ill-being. Rather, the finding is of a relational unfolding of how the geo and people mutually co-develop and affect one another. Putting it simply, people are clearly ‘made’ by the geo at the same time that they ‘make’ it in return, in which regard an emphasis on what people do in, with and through spaces—including how they actively ‘place-make’—is definitely part of what our review has disclosed.

In Table 3 we show the ‘Kinds of Places’ identified, providing thumbnail accounts of what each one implies and also specimen quotes from the literature to exemplify something of what is involved in each case. Throughout our tables we retain—where names are given (real or pseudononymised) in the source papers—these named identities of the voices of experience in order to recognise the importance of their agency and personhood. We also retain the original punctuation (or lack of it). In Table 4 we show the ‘Affective Qualities’ identified, providing brief explanations for what each one delimits and, again, some specimen quotes from the literature (some with explicit reference to the geo, some without). Tellingly perhaps, the prevailing ethos of what we listed on both sides of our pinboard was negative, evoking distressed, sometimes deeply ‘dark’, overarching affective states (‘insecurity’, ‘pain’, ‘loneliness’, etc. [cf. ‘relaxing’]) as evidently bound up with places cast in a predominantly negative vocabulary (‘broken’, ‘lost’, ‘disrupted’, etc.). There are potent prompts here to wondering what is at stake in the damaged attributes of places or, better, in how and why places may become perceived as both damaged and damag*ing* to psychological well-being. In the ‘Kind of Places’ column, however, there are also hints about places cast in a more positive vocabulary, with nods to places enabling ‘safety’, retreat’ and ‘launching’, alongside the possibility of places being ‘idealised’—idealising what would be mentally soothing and enriching places – and soaked with ‘imagination’ and ‘fantasy’ (although the latter pair could of course instigate fears and terrors as much as hopes and pleasures). There are further prompts here, therefore, for pondering what might indeed be improved places for well-being pictured by voices of experience, both as found in the wider social world and with respect to quite specific ‘enabling places’ (Duff, 2012)—places of sanctuary, gathering, mutuality, advice, advocacy and more—that might be conceived and even one day realised. There are also intriguing challenges made to usual presumptions about places that are deemed ‘safe’ and how control is enacted in these places, particularly in relation to acts of ‘self-harm’ and suicide, demonstrating the importance of being attentive to the nuances and intricacies of individuals’ relations with place.

**Table 3.** ‘Kinds of places’ (based on analysis/workshop 28/06/2024).

Label^a^	Thumbnail descriptor	Exemplar quotes
Broken places	Referring to systems and structures that limit and constrain aspects of everyday existence.	They made me go on JSA and I began to get psychotic again, really ill. When they changed from IB to ESA I went to the interview and they denied it so I had to go on JSA. So I went to sign on every week and you have to keep a record of all the jobs you’re looking for and apply for anything and take it back to the interview once a week. So I was doing that and the stress was building up and I started hearing things, and seeing things. When I get very depressed and stressed I start hearing things and seeing things. (Christine, in Lowe & DeVerteuil, 2020a, p. 4) You know what’s really killing us? We’re economically shagged. You can’t go nowhere, you can’t get nowhere. That’s what it is. All these things will just keep escalating. It can’t change. We cannot get no better, and that’s what’s happening. (Anonymous, in McLean et al., 2003, p. 666)
Disrupted places	Highlighting places where aspects of self and/or living are (often negatively) altered.	I would say this place [forensic hospital unit] has amputated my sexuality. Definitely, it’s—it’s not my home, it’s not—it’s not a free environment and . . . it’s a—it’s so anti-life. (Anne, in Brown & Reavey, 2019, p. 131) After my wife picked me up from the hospital, things began to get really weird. As soon as I got home, I drove off to have breakfast with my friend Frank. After breakfast, I drove around and noticed concentration camps all around. What I actually saw were schools getting major remodelling done. There were chain link fences all around them to provide a safety barrier. That is why I thought they had the appearance of a concentration camp. While I was driving and while eating at the restaurant, I felt Hitler’s atrocities were still going on, and kids were being marched off to concentration camps to be executed. At the restaurant, I thought the hamburger patties were made from human flesh. I thought that I had to eat the human flesh burgers to show my allegiance to Hitler. (Mariant & Mariant, 2006, p. 53, in Chouinard, 2012, p. 147)
Entrapped places	Denoting places where there is a sense of a lack of freedom to move or to get out.	I didn’t have any escape. (Gareth, in McQueen & Henwood, 2002, p. 1550). My mental illness was a straight jacket; my voice was crushed. I was silenced. (McMahon, 2023, p. 10)
Idealised places	Relating to specific places where positive feelings, desires and aspirations are held.	It’s become my sanctuary, a place just for me, for cooking, for trying out new things but also I guess just for the way it makes me feel. Like, pulling into the driveway, it’s like entering another world for me. I know that when I get into the kitchen and make a cup of tea it will be me here, that’s me and then the outside world. It (the kitchen) just makes me feel safe and in control of things. (April, in Duff, 2012, p. 1393) I love the gym I love the gym [. . .] it’s a place where no-one knows who I am [. . .] but I can just sort of be [. . .] I like I even like using the shower there and that kind of thing because I really hate being at home and anything that I cannot do at home [I: mmm] is really good so I really I like showering there and doing my hair there and you know [. . .] just you know eating lunch there I just really like the I really like the space [. . .] I guess this is a space that I don’t mind being mine [. . .] I don’t I don’t have any issues about being a member of a gym [. . .] it’s like I feel normal there I feel like I’m not in an institution [,] I feel like I’m not special. (Lou, in McGrath & Reavey, 2013, p. 128)
Imaginative places	Signalling to places that exist beyond everyday realities that draw upon fantasy, delusion and imagination.	‘[h]e described more than one imaginary scenic place: a good-time landscape with green meadows and blue lakes; a bad-time landscape with a night-moon landscape; and a peace-calmness-relaxation landscape incorporating a savannah landscape with pastel shades, indicating a desire for peace, calmness, a chance to breathe, and a view into the distance—a compensation for his painful experiences. (Client 2, in Lengen et al., 2019, p. 28) A forest looms inside my mind, memories in rows. The shadows cast of white bones and headstones, trees marked, stark and waiting, cut into survivor-ship masts. This pain waits for voice, encased in darkness, silt, and silence. This bodymind carries volcanic scars restraint, confinement, self-starvation, blood, violence comas, restarted heart, hospital gowns and locked doors, grinding shut. I write to an unknown audience across a sea: these are a few of my dangerous dreams. (Elwyn, 2022, p. 1)
Landing places	Referring to places where you arrive after (or during) a difficult journey, often connected to (sometimes precarious) feelings of safety (Larsen and Topor, 2017).	I think the place to start would be when I was at rock bottom. I was talking to a wall, talking to my cigarettes, listening to my voices . . . I stayed in my room . . . [with] that out of control fire that was within me. (David, in Liggins, 2018, p. 663). I feel like I’m kind of disappearing, that I’m no longer being, so just seeing the blood it kind of reminds me that I am here [. . .] obviously my heart’s still beating because the blood’s still coming out and it’s just nice to just see firm evidence that I am alive. (Emma, in Black et al., 2014, p. 79)
Mobile places	Connecting to places that are travelled through.	Looking back over over a period of years my tendency is to go out to be walking to be quiet to be quiet feeling I’ve I’ve got to be moving and not being in one place as a sort of anxiety maybe and a sort of claustrophobia type thing I mean I know I know a few years ago I was when I was having crises I I would have this very strong feeling that I that it it say late in the evening that I’ve got to go out and at the same time I would kind of know that its not a good cos I’d but this kind of claustrophobic feeling is kind of I’ve got to I’ve gotta get out of my flat I’ve got to go somewhere I’ve got to do something um and usually that wins out. (Bryan, in McGrath & Reavey, 2015, p. 119) Going for a walk at three o’clock in the morning, I feel quite safe on the street, ’cause it’s dark and there’s no one around, everyone else is asleep. There’s no energy floating around the air, you know people’s manic energy, everybody’s resting. So I find the streets around here quite calm and peaceful at night, even though it goes against what should be because you’re not supposed to feel safe at night. I don’t know but for me the darkness is safe. The world is at rest and it just makes me calmer. (Matt, in Duff, 2012, p. 1393)
Retreat	Referring to places that feel restorative, often connected to particular types of environments.	I have always loved the Botanical Gardens, even as a child, but ever since I was diagnosed it has just been so important for me. It just makes me feel alive again, the greenery, all the plants, all that life all around. And the silence too, like I can just sit and watch things go by. It just makes me really happy. (Liz, in Duff, 2012, p. 1392) By the sea. Seriously, by the sea. It’s probably not the answer you wanted but it’s by the sea. That’s where I grew up, that’s where I belong. That was just heaven. Just the wind, the sand. It’s just total relaxation for me. It’s an escapism. (Ryan, in Ahmadu et al., 2021, p. 3)
Safe places	Signalling places where a sense of security can be found.	Well it’s my place, it’s peaceful, secure, a really good feeling you know ’cos I have lived rough in the past. It’s different here, where I have my DVD player and my radio and stuff, so I don’t have to go anywhere. I’m better now that I have a place to call my own, it’s an anchor for everything else in my life. (Ric, in Duff, 2012, p. 1392) [In hospital] I had some time to think about things and put things into perspective. [. . .] I feel safe. (Ryan, in Wyder et al, 2016, p. 340)
Stolen places	Referring to places that are no longer easy to access or be accepted in.	You ask me what is harmful to my recovery, I’ll tell you again loud and clear, it was when they did not allow me to go outside, to go in the park, to walk near the water or let me take walks. Even if they had followed me with a syringe . . . how much would it cost to allow people to go walk outside? They could hire people to do just that: accompany people on walks. (Women in early-40s, in Bouchera et al, 2019, p. 65) It is hard at first when you go to these places as you feel like a misfit, because you have bipolar, Asperger’s and things, you know, and it is a big step to go somewhere. (Andrew, in Cogan et al., 2021, p. 361)

a In alphabetical order to signal that no necessary priority should be given to any one entry or assumption made about its relative numerical prevalence in papers located by the literature review.

**Table 4.** ‘Affective qualities’ (based on analysis/workshop 28/06/2024).

Label^a^	Brief explanation	Exemplar quotes
Control	Referring to feelings of power relating to the occupation and use of space and place.	A 17 year old college girl sat on the ledge of her window on the second floor of the dormitory. It was pitch dark and no one saw her sitting there, including those in the nearby dormitory room. For a long time, she sat there thinking about jumping and flying and vaguely about killing herself. Several times, she wondered whether anyone would come by and notice her there. For what seemed like hours, nobody did; she jumped. . . . At the time she jumped from the window ledge, she fully believed that she might fly and did feel while falling a distinct sense of control over both her body and the environment. (Rothenberg, 2006, p. 17) An example of how I was not permitted to have any agency, as is the case historically for mental health inpatients, is when I wasn’t allowed to eat my breakfast in the courtyard. The nurse said to me, “You have to eat at the dining table!”. I quietly asked “Why?” She stated, “Because I said! It is policy!” “Why?” I asked. “Just because!” she yelled. (McMahon, 2023, p. 11)
Insecure	Connecting to feelings of being outside of the known and the comfortable.	I always felt quite on edge. (Donna, in Lowe & DeVerteuil, 2022, p. 452) Kitty, a twenty-five year old middle child in a family of nine children, lives on a small mountain farm which she describes as ‘wild and lonesome.’ Although a bright student, Kitty dropped out of school because she found it ‘boring.’ Home, however, provided even fewer outlets, and Kitty’s behavior rapidly deteriorated. She spent her days eating, sleeping, and staring out the window. She has been hospitalized five times for involutional melancholia and suicidal preoccupations. Recently, Kitty has begun to hear the voices of saints who tell her to ‘get out’ and ‘go away.’ But Kitty doesn’t know where to go. (Scheper-Hughes, 1978, p. 69)
Invasion	Referring to feelings of fear and disgust over unwanted intrusion into physical and psychological space.	Jane was intensely distressed by being forced into physical contact with other bodies, coats, bags and exhalations whilst travelling on a crowded bus, and was immensely worried that she would contract some form of illness. On leaving the bus she became convinced that she had stepped in dog excrement on the pavement, and was overpowered by a sense of invasion and contamination. Reaching the safety of home, she stopped in the hallway, scared of entering the rooms of her house for fear of contaminating everything she touched. Taking a pair of rubber gloves from her shopping bag she cautiously removed her shoes and clothes, placed them in a bin bag, and wept uncontrollably. Unable to cook the food that she had bought from the supermarket because it had been contaminated, she retreated to bed. (Segrott & Doel, 2004, pp. 602–603) I was in seclusion and I was in there for days. And in between that I, there were parts where I was brought out and strapped to a bed. But I was really—they had to use security a few times to hold me down to put—to give me a needle and I just had bruises all over me. (Geraldine, in Stone et al., 2020, p. 67)
Loneliness	Relating to feeling alone and unseen.	I just felt totally alone and totally lonesome and helpless as well. (Brendon, in Holton et al., 2023, p. 1763) People see the diagnosis and they don’t see the person, and you don’t feel validated. (UB9, in Jones et al., 2009, p. 635)
Longing	Connecting to feelings of escape to somewhere else, often away from (internal and external) places of acute difficulty.	I live in Zone 1 in Central London, I have no choice but to be in crowds. I would love to be ‘far from the madding crowd’. I would love to be back in Scotland where I grew up in a small village. I would love to be back there. This is why I call myself a prisoner. I don’t have the economic means to change my life. I don’t have the opportunities . . . I’ve given up on life. (Liam, in Lowe & DeVerteuil, 2020b, p. 5) Not only do my experiences with bipolar and OCD lead to intrusive thoughts and chronic suicidal ideation that (especially when in the midst of the complexities of a mixed state) involve repeated visualizations and deeply embodied yearnings to soar off my roof into the ether and/or plummet to the ground below. (Cosantino, 2023, p. 4)
Painful	Signalling to difficult feelings of loss that fracture experiences of space and place.	I said of my illness that “my world would fall apart; I would fall apart’”; others detailed being: scattered, shattered, smashed and broken. (Author, in Liggins, 2018, p. 663) And some of that is my fault, I suppose. Should I have pushed, should I have dragged him off to places, should I? - but you couldn’t do that, he just absolutely refused . . . I was afraid that I would lose him altogether. (Larissa, carer in Stone et al., 2020, p. 67)
Relaxing	Referring to feelings of calmness and peace in and relating to particular places.	CR: What are good places?. . . Sam: The park . . . We sit up there amongst the trees and listen to our little portable radio that runs by batteries . . . CR: Why do you go up there?. . . Sam: Spaceful. Peaceful . . . Time out. It gives me time to think about things . . . . (Sam, in Robinson, 2005, p. 55) [T]hat’s the street [running parallel] to the corner of my balcony . . . this is where I often take the bus to go anywhere and everywhere in the city. This is my place of . . . I see it from my balcony . . . it calms me. It makes me feel good to say: “Ok I’ll go to my balcony and I’ll contemplate the world passing by, suntan, breathe fresh air.’”. It’s the view that’s calming, a direct view from my balcony. (Pierre, in Piata et al., 2017, p. 76)
Sadness	Connecting to experiences and memories of places that feel heavy and difficult to revisit.	With having mental health issues, your mood can be all over the place so you need something to like counteract whatever you’re feeling . . . if you walk, even walk through like parks and you look at the playgrounds that haven’t been done up in twenty years, and everything’s falling apart, it makes some places that should be happy more miserable. (Euan, in Birch et al., 2020, p. 7) “Why else would I be here?” Shafeeqa asked me indignantly, one cold December day in 2010, while we warmed our hands in front of the gas stove (chula) in the small, dark staff room. I had asked Shafeeqa if she used to come to the hospital [Government Psychiatric Diseases Hospital] as a child. She nodded, “I used to come here, yes. But I try not to remember those days. Those days, things were much worse. The patients were chained from head to foot.” After a pause, she continued, “but then the [Supreme] court order came, and then they stopped chaining.” (Shafeeqa, in Varley & Varma, 2018, p. 632)
Trapped	Referring to uncomfortable and distressing feelings of being in place where there is a lack of possibilities to get out.	I can’t stand living here and I have no prospect of moving. For me, my flat is a prison. The only reason why I am not dead is that there are no ligature points in my flat. I am so unhappy there. (Liam, in Lowe & DeVerteuil, 2020a, p. 5) Psych call [this place, St Elizabeths Hospital, Washington DC, US] a prison . . . Some say it ain’t [but] all I can see is bricks and wood and wires and iron. (Abraham Tibbs, inmate of this asylum, 1911, in Gambino, 2008, p. 398)

a In alphabetical order to signal that no necessary priority should be given to any one entry or assumption made about its relative numerical prevalence in papers located by the literature review.

What this exercise instructively demonstrated (see Figure 1) is that there is no straightforward relationship between the grounded spaces on our base map, on the one hand, and particular projections or affectivities listed on the right and left of our pinboard, on the other. To put it another way, just as home or hospital could be the subject of the most intensely negative (dispiriting) or positive (uplifting) of experiential responses, so the same is true of any other space or place that might be recognised and mapped. In Table 5 we categorise a large number of such quotes according to their space or place of reference, including some attributed to nameable settlements or regions and also ones where the referent is arguably more imaginative, metaphorical or delusional (implying a whole other body of questions that might be asked about the voicing of the geo from below).

**Table 5.** Grounded places and spaces (based on workshop 28/06/2024).

Category of place/space	Thumbnail descriptor	Exemplar quotes
*Self*	Relating to aspects of place that are often perceived and/or experienced as internal.	. . . the nooks and crannies, those places of emptiness within the shell that was my skin. (Author, in Liggins, 2018, p.663) My inner landscape is a landscape that is actually real, I mean, I have been there . . . . (N, in Barbieri & Rossero, 2024, p. 8)
*Home*	Depicting spaces and places that relate to associations of dwelling.	I like being at home it’s my sanctuary [I: mmm] it’s where I feel safe you know I can have who I want in my house and you know chill out do what I like and feel relaxed in myself. (Janet, in McGrath & Reavey, 2015, p.121) She did not want [community mental health] staff to make home visits, thinking that neighbours would gossip and call her crazy. (Family member, in Guvenc & Oner, 2020, p. 712)
*Healthcare*	Signalling to placed experiences where forms of medical interventions are given.	How gross, with an open door to the hall where elderly people sat waiting for their consultation with their GP, with endless time to stare at us, a bunch of exhausted ‘junkies’ standing in a corner queuing for our pee. Oh my God, please! This just wasn’t for me! (Asta, in Hope et al., 2023, p.4). Nurse begins to search the estate, holding up artefacts one by one. “Is this what you want? I’ll confiscate everything you love!” Her hands stumble across a hidden scalpel and broken glass. “Oh.” “Oh dear”. Says psychiatrist. “We’ll need to document that.” Nurse swallows, pats the body’s hand. “It’s okay sweetheart. Maybe when you’re feeling a little less . . . distressed you can talk to us.” Night passes. Body is visited by gravediggers, torches glowing over its bones, searching for signs of life. Resting place desecrated, bones rolled in effort to shake them awake. Morning comes. Psychiatrist tells body if it doesn’t come back to life, it will be taken away and shocked into activity. Deep in the soulchamber, the tiny seed is curled and soft. It rocks itself. (Elwyn, 2024, p.5)
*Institution*	Referring to historical and contemporary spaces and places of incarceration relating to mental health, including asylums, mental hospitals and prisons.	. . . if I’m distressed sometimes I will actually surface in the grounds of the psychiatric hospital because it’s safe . . . the ward wouldn’t feel safe to me . . . but actually being in the grounds in a sense has a sense of safety in that I know there are people sort of around that understand me which I don’t feel like if I went into town I wouldn’t feel anything like that . . . . (Julie, in McGrath & Reavey, 2015, p. 120) Her eyes light up. “Who told you?” she asks me eagerly. The happiness in her voice strikes me. The fact that I received this news suggests that she exists within a network of social relations; she is remembered in an amnesiac space. We walk, arm-in-arm, back toward the closed ward where she lives with 21 other women. Her joy is palpable. Various hospital staff see us and call out her name, as if afraid that she might run away again, as if saying her name out loud will affirm her presence. (Nusrat, in Varley & Varma, 2018, p. 635)
*Post-asylum spaces*	Connecting to the diverse spaces and places that comprise the deinstitutionalised landscape of mental health care, including drop-in clinics, GP surgeries and community centres.	We arrived in these places distressed, struggling with illnesses that we did not understand; disconnected and fragmented, we had lost ourselves. In response to these experiences, a place that was healing was one that offered safe haven, (metaphorically) holding us enough in a collaborative environment of care, hope and trust; while providing or creating space and opportunities for the hard work of exploration that underpinned our healing. (Liggins, 2018, p.665) [I]t’s really quite scary because it’s just you know that there’s some really disturbed people come into this place and sometimes they’re really smelly . . . you just never know what you’re going to come across and there’s a funny smell there as well and just the way that the other people are barriered you know barred off from you [I: mmm] like you’re the plague I dunno I just really dislike it I think it’s an awful space to wait um it’s just like nobody really cares . . . it feels really like you know we’re not worthy of a decent space you know [I: mmm] it’s like this waiting room with these ancient magazines. (Lou, in McGrath & Reavey, 2013, p. 127)
*Public places*	Signalling the range of spaces and places that can be accessed by a wide range of people and communities and exist in public space.	I said to you about coming here [a café in an art gallery] and how it gave me a sense of serenity. There’s something about the color, and the order, and the quality of light, and the finishes are quite smooth, and it makes me feel something inside my body when I’m in these environments. But in my flat, it just looks as if someone’s got a skip and emptied it through the roof (Harry, in Lowe & DeVerteuil, 2022, p. 453) [Loneliness] is more intense when I am physically alone . . . even more so at night because I am not around people and unable to reach out to people. Sometimes I’ll go to church or read in a coffee shop just so that people are there. Being around people definitely helps. (8013, in Ludwig et al., 2022, p. 546)
*Community spaces*	Referring to places that are created and run specifically to support mental health communities.	[This place] sparks that hope, like, you come in the door a really broken woman . . . I didn’t think my world was ever gonna get any better than that. Erm, and coming here, the staff, the students, the facilitators, erm, they all showed me that actually hope is, there is hope. Erm, when you find that spark of hope, er, it just starts as a little, little fire, a little flame and then before you know it, it’s, it’s a full-on raging fire. (Deborah, in Claisse et al., 2004, p. 9) I think this project is about giving people an environment where they can regain their confidence . . . It’s more than just a physical space. You could reproduce this workshop, you could put the same number of people in it, but if you haven’t got an understanding of what makes the heart of it beat then it’s not going to end up being the same thing. (Brian, in Smith, 2021, p. 158)

## Discussion

Our objective has been to nuture insight into the possibilities for a geopsychiatry from below, conveying the groundwork required for considering voices of experience in relation to mental ill-health and the geo. This paper hence acts as a starting point for extending geopsychiatry’s remit, spirit and purpose, offering an important window into the lived worlds of mental health, both in terms of the manifold complexities of the world that it illuminates and its countless localised ‘worldings’ across the endlessly diverse spaces and places of the geo. Our inquiry has explored—if in an inevitably preliminary fashion—the complex connectivities between grounded places, how these places are experienced, notably in terms of the emotionally-charged responses and (often vulnerable) affective states of the people concerned, which could be long-turn or episodic. The upshot is what we call a framework, a prompting to take seriously how the elements identified in Table 2 dovetail with one another, which is then amplified by the labels and categories suggested in the left-hand columns of Tables 3 to 5 as well as being fleshed out by the richly textured details of the exemplar quotes given in the right-hand columns of the same tables. We do intend both the former and the latter to be prompts for other researchers’: prompts regarding what generalisations might be advanced about the relations between place and mental (ill-)health, but also prompts about the delicate particularities of the geo—its physical, social, symbolic, imaginative, performative and countless other facets—that, we contend, have such a profound, if rarely foregrounded, impact at the heart of mentally unwell and distressed lives.

We recognise that our claims are a geo-inflected microcosm of much broader, often contentious, debates about the standing of psychiatry as academic discipline and clinical practice. Such debates have been ongoing since a formalised speciality called psychiatry—or ‘alienism’ or ‘mental science’—first clearly took shape during the nineteenth into the early-twentieth centuries. They intensified from the 1960s during struggles around ‘anti-psychiatry’ and then from the 1970s when service user and psychiatric survivor movements began to question the constructs, institutions and practices of biomedically-inclined psychiatry. Marxist, feminist, postcolonial and the decisive Mad Studies challenges have been added into the mix, alongside the call for psychiatry to look beyond the hallways of quantitative (experimental, hypothesis-testing, random-control trials-dominated) scientific inquiry toward forms of qualitative inquiry wherein matters of voice, experience, sensation, feeling, hopes and fears become centralised. Our geopsychiatry from below has moorings in all these critical lines, but we also suppose that there is something encouraging about how a corner of psychiatry—this new geopsychiatry—that is opening psychiatry to the disruptive complexities of place may also act as a gateway for considering the many-sided challenges of people’s lived experience in place.

## Conclusion

Paying attention to the fragmented and marginalised voices of experience through our review process highlights the possibilities for new ways of understanding what matters to people and why as they live in and through the worlds of mental (ill-)health, lending crucial insight into where attention may be required to create and sustain more caring and hopeful worlds, not just for those with mental ill-health but arguably for all of ‘us’. Such prompts are ones that we are now taking into a more action-orientated stage of our research, reported in McGeachan et al. (2025), wherein we have undertaken a further workshop—with a mixture of experts by experience and mental healthcare practitioners—to road-test interactive ‘geostory-telling’ and also to furnish materials for what we have now created as a ‘Geo-Story Resource Park’.^10^ The hope is that others – maybe researchers but potentially anyone involved in working alongside people with mental health problems—might experiment with running geostory-telling exercises, not necessarily with any definite therapeutic aims in mind but, rather, just as something interesting and stimulating to do (for all concerned). There are hence academic, therapeutic, professional and practical imperatives for why a geopsychiatry from below is needed *and* why at least some contributions to that project should be attendant to the ‘from belowness’ of the geo as centralised in our contribution.

## Notes

1 We acknowledge that the phrase ‘from below’ does *itself* risk reinforcing a sense of there being a hierarchy of more-or-less valued knowledge about psychiatry. As one reviewer notes, many people ‘would not want to be characterised as ‘from below’.’ Nonetheless, we elect to continue using the term given its evocative and perhaps provocative aspects.

2 As one reviewer sagely clarifies, our move here dovetails with a postcolonial critique wherein, quoting our reviewer, ‘Global mental health has been criticised for being top-down, almost colonialist in blanketly imposing ideals and aspirations onto all regions of practice’ (see also Beresford & Rose, 2023).

3 Ruth Kirkpatrick (see https://tracscotland.org/storytellers/ruth-kirkpatrick/) is a professional ‘story-teller’ with a particular interest in the therapeutic role of stories in assisting people through trauma. She was introduced to McGeachan by previous research collaborators when we were seeking expert input for the present project.

4 Morag Macgilchrist and McGeachan have collaborated together for several years on community arts projects. Morag self-describes as follows: ‘I live and work in Glasgow but was raised on the Isle of Arran. I practice at Project Ability, a Glasgow -based arts charity. My work explores my mental ill health and my special interests of dolls, superheroes and anime.’

5 All papers from which we have taken extracts/quotes are included in the References. We have not created a separate list of all papers identified in our review: to have referenced all those identified in the ‘expansive’ search would have been meaningless, while virtually all those identified in the ‘narrower’ search are ones from which we have lifted extracts/quotes and hence appear in the References anyway.

6 We acknowledge that selection of extracts/quotes was a subjective process, anchored in the hermeneutic relation between reader (us) and text being read, but our own positionality as experienced/informed researchers offers at least some assurance that our selections will be appropriate—and not somehow misleading—for the specific purposes of the present exercise.

7 We should underline that these two headings (‘Places’ and ‘Qualities’) are very much intended as place-holders, and we envisage that we, and maybe others who build on this work, will subsequently revisit, revise and expand them. We might add that a considerable amount of thinking—bound up in the longer-run philosophy and social theory of academic human geography and cognate disciplines (e.g. Cresswell, 2014; Merriman, 2022)—is germane to how we understand these elements, even if their exact formulation here emerges *from* our review.

8 This was more a schematic mapping of types of space than one representing an actual neighbourhood, but there is no reason why a running of this exercise could not operate with a base map representing a specific concrete place, including natural environments or landscapes, or extending its scalar logic to whole settlements, regions, nations or even the likes of tourist destinations.

9 The papers that we identified were based chiefly but far from exclusively on voices from experience from the Global North: a fuller extension of the approach taken in this paper could usefully attend more critically to the geography *of* these voicings (as one reviewer prompted).

10 Printed copies available from the lead author at her University of Glasgow address. We call this phase of our endeavours ‘geopsychiatry-in-action’.
